# Supplementary material for: Autophagy-mediated apoptosis eliminates aneuploid cells in a mouse model of chromosome mosaicism
Source: Nat Commun. 2020 Jun 11;11:2958. doi: 10.1038/s41467-020-16796-3 (PMC7290028; doi:10.1038/s41467-020-16796-3)
Supplement: Supplementary file 1 — Supplementary Information [file 41467_2020_16796_MOESM1_ESM.pdf]

Supplementary Figures

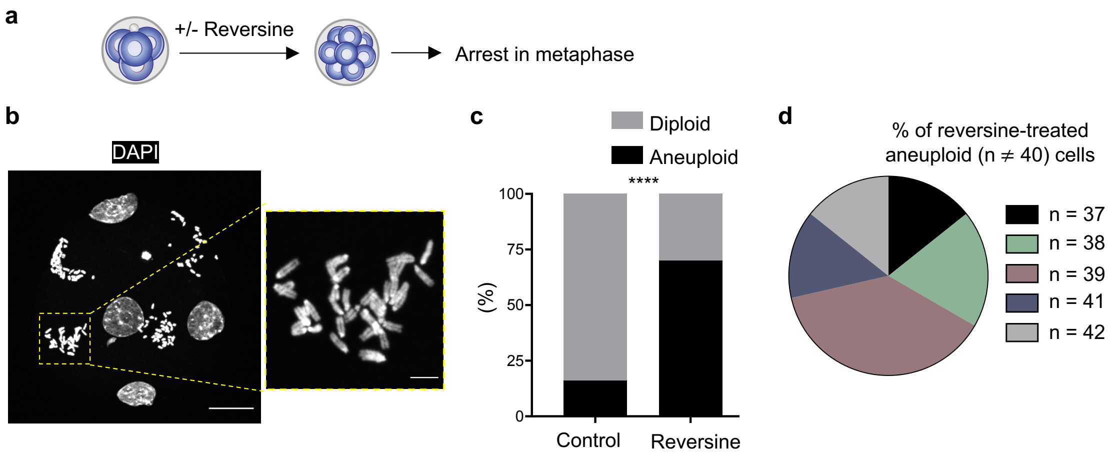

Supplementary Figure 1. Increase in aneuploidy following reversine treatment of mouse embryos. (**a**) Embryos were treated at the 4-8 cell stage with reversine or DMSO. They were arrested in the metaphase at the 8-16 cell division and chromosome spreads were performed. (**b**) A representative image of a chromosome spread for a reversine-treated 8-16 cell mouse embryo. The number of chromosomes = 37, indicating an aneuploid cell. Scale bar, 30 µm. Square indicates the magnified region (individual cell). Scale bar, 5 µm. (**c**) Number of chromosomes per cell were assessed by chromosome spread. If the number of chromosomes = 40, the cell was categorised diploid and if the number of chromosomes ≠ 40, the cell was categorised aneuploid. Fisher's exact test and ****p < 0.0001. (**d**) Distribution of reversine-treated cells that were categorised aneuploid in (**c**) according to the number of chromosomes (n). For (**b**), (**c**) and (**d**), Control n = 38 cells, reversine-treated n = 30 cells. Source data are provided as a Source Data file.

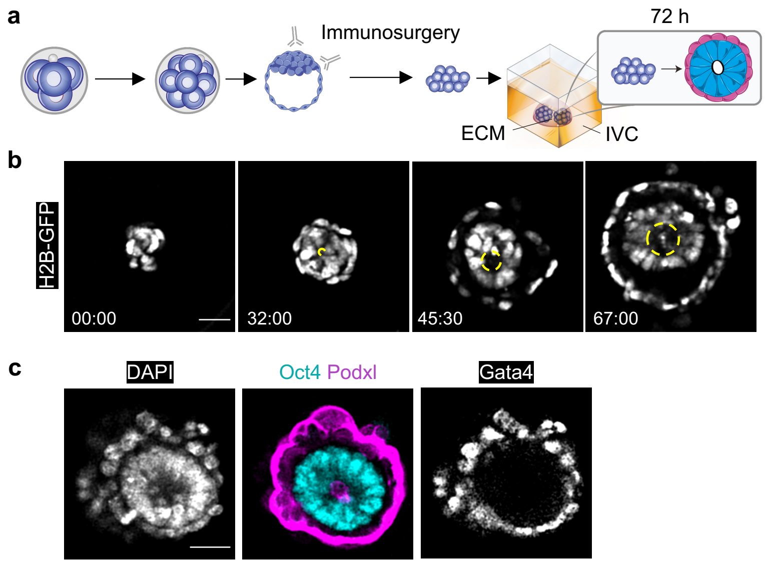

Supplementary Figure 2. Epiblast remodelling at peri-implantation development using *in vitro* model. (**a**) Embryos were cultured from the 4-cell stage until the late blastocyst stage when immunosurgery was performed to isolate the ICM from the TE. The ICM was embedded in Matrigel and cultured in IVC medium for 72 h to allow its development into an epithelised EPI surrounded by a layer of PE with a central lumen. (**b**) Sequential representative images of a time-lapse series of a Histone H2B-GFP-expressing ICM, cultured according to the schematic shown in (**a**) (hour:minute). Yellow line marks the site of the emerging lumen. Scale bar, 30 µm. (**c**) ICMs cultured according to schematic shown in (**a**) for 72 h were analysed for the EPI (Oct4 positive) and PE (Gata4 positive) characteristic post-implantation rosette morphology and presence of a lumen (n = 16/34 organized structures after 72 h of culture) . Scale bar, 30 µm. For (**b**) and (**c**), n = 16 organized structures.

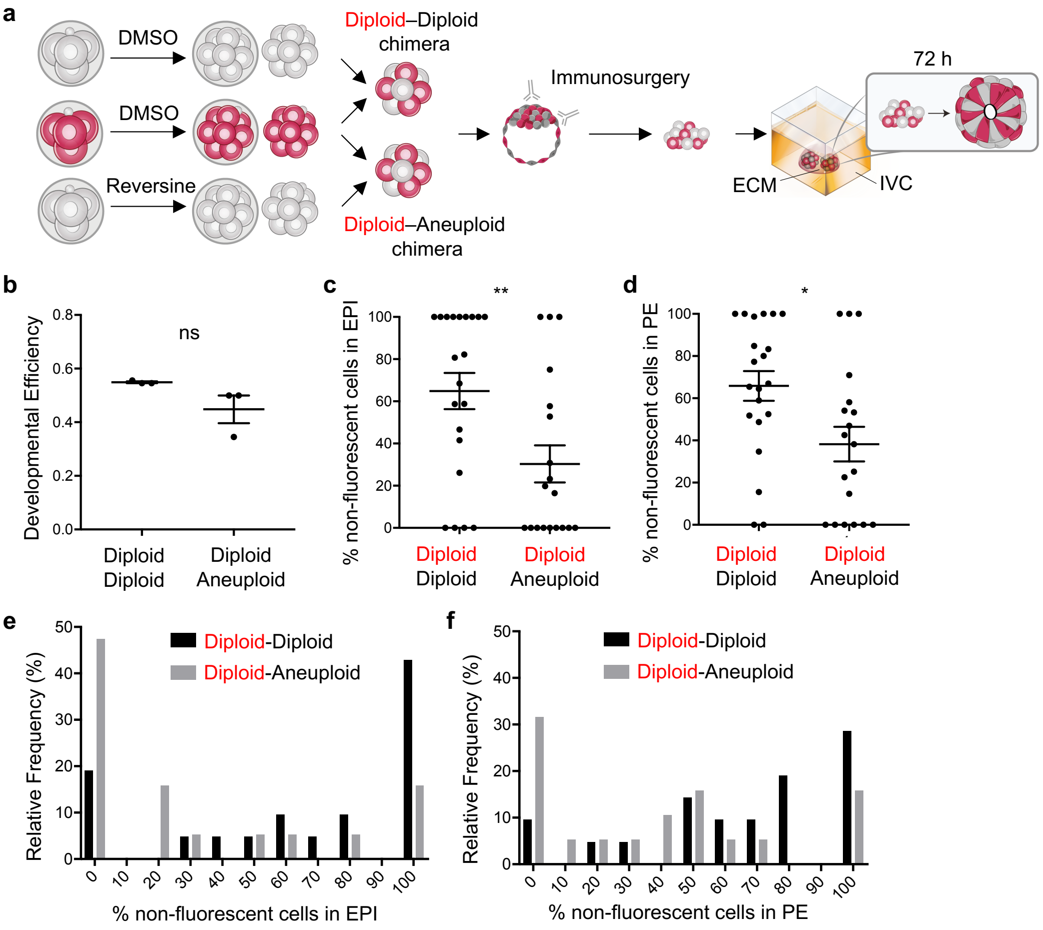

Supplementary Figure 3. Aneuploid cells become eliminated during epiblast remodelling at peri-implantation development *in* vitro. (**a**) Embryos were treated with reversine (or DMSO) at the 4-8 cell stage transition and 8-cell chimeras containing a 1:1 ratio of control (diploid) and reversine-treated (aneuploid) cells were constructed from mT/mG (red) diploid cells and non-fluorescent aneuploid cells at the 8-cell stage. Immunosurgery was performed at the late blastocyst stage. The ICMs were embedded in Matrigel and cultured in IVC medium for 72 h. (**b**) The efficiency of chimeras in forming an epithelised EPI surrounded by a layer of PE with a central lumen after 72 h *in vitro* culture is equivalent for diploid-diploid controls and diploid-aneuploid chimeras. n = 3 independent experimental groups. Mann-Whitney test and ns = not significantly different. After culture, each chimera was assessed for the percentage of non-fluorescent cells to total number of cells in the EPI (**c**) and PE (**d**). Frequency distribution of non-fluorescent cells in the EPI (**e**) and PE (**f**) of control diploid (red)-diploid (non-fluorescent) chimeras and of diploid (red)-aneuploid (non-fluorescent) chimeras. For graphs (**c**) and (**d**), Student's t-test, *p = 0.0145 and **p = 0.0077. For graphs (**b**), (**c**) and (**d**), all data are mean ± s.e.m. For all the graphs, diploid-diploid n = 21 chimeras; n = 2636 EPI cells; n = 3003 PE cells and diploid-aneuploid n = 19 chimeras; n = 1900 EPI cells; n = 2306 PE cells. Source data are provided as a Source Data file.

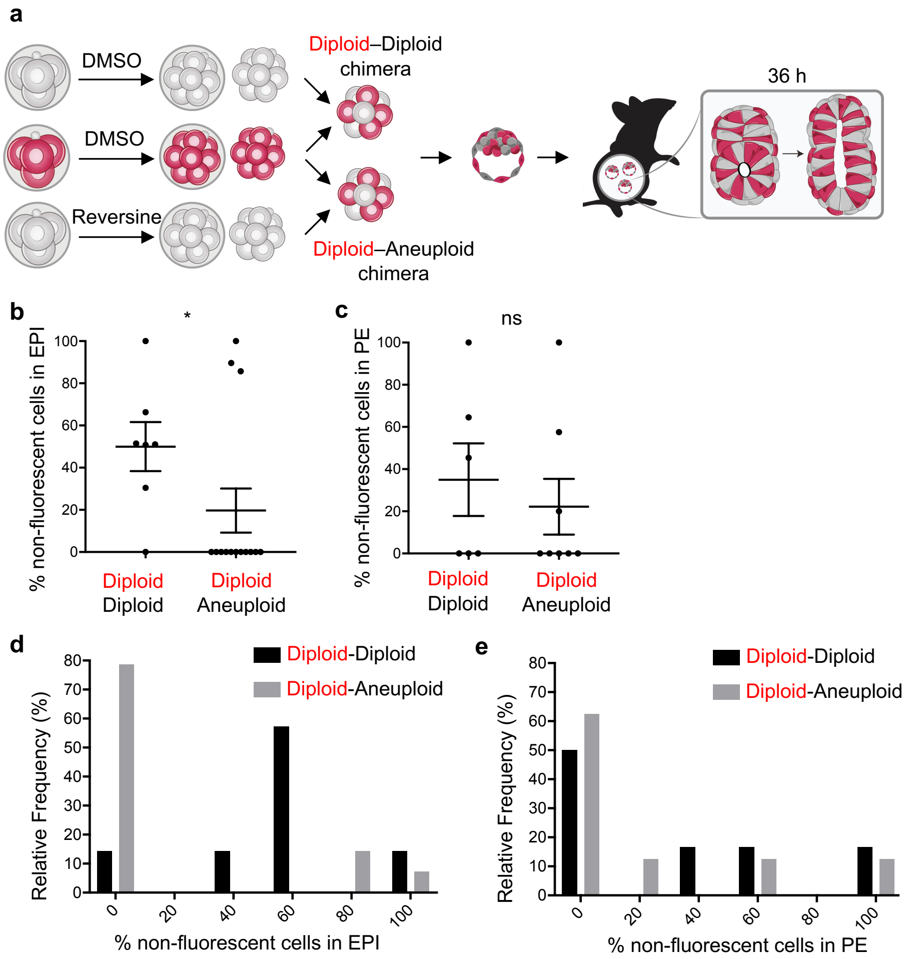

Supplementary Figure 4. Aneuploid cells become eliminated during epiblast remodelling at peri-implantation development *in vivo*. (**a**) Embryos were treated with reversine (or DMSO) at the 4-8 cell stage transition and 8-cell chimeras containing a 1:1 ratio of control (diploid) and reversine-treated (aneuploid) cells were constructed from mT/mG (red) diploid cells and non-fluorescent aneuploid cells at the 8-cell stage. At the early blastocyst stage, chimeras were transferred to pseudo-pregnant mothers and recovered 12 h after implantation and cultured in IVC medium for 36 h. After culture, each chimera was assessed for the percentage of non-fluorescent cells to total number of cells in the EPI (**b**) and PE (**c**). Frequency distribution of non-fluorescent cells in the EPI (**d**) and the PE (**e**) of control diploid (red)-diploid (non-fluorescent) chimeras and of diploid (red)-aneuploid (non-fluorescent) chimeras. For graphs (**b**) and (**c**), Mann-Whitney test, $*p = 0.0370$, ns = not significantly different and all data are mean ± s.e.m. For graphs (**b**) and (**d**), diploid-diploid n = 7 chimeras; n = 807 EPI cells and diploid-aneuploid n = 14 chimeras; n =1640 EPI cells. For graphs (**c**) and (**e**), diploid-diploid n = 6 chimeras; n = 1110 PE cells and diploid-aneuploid n = 8 chimeras; n = 1488 PE cells. Source data are provided as a Source Data file.

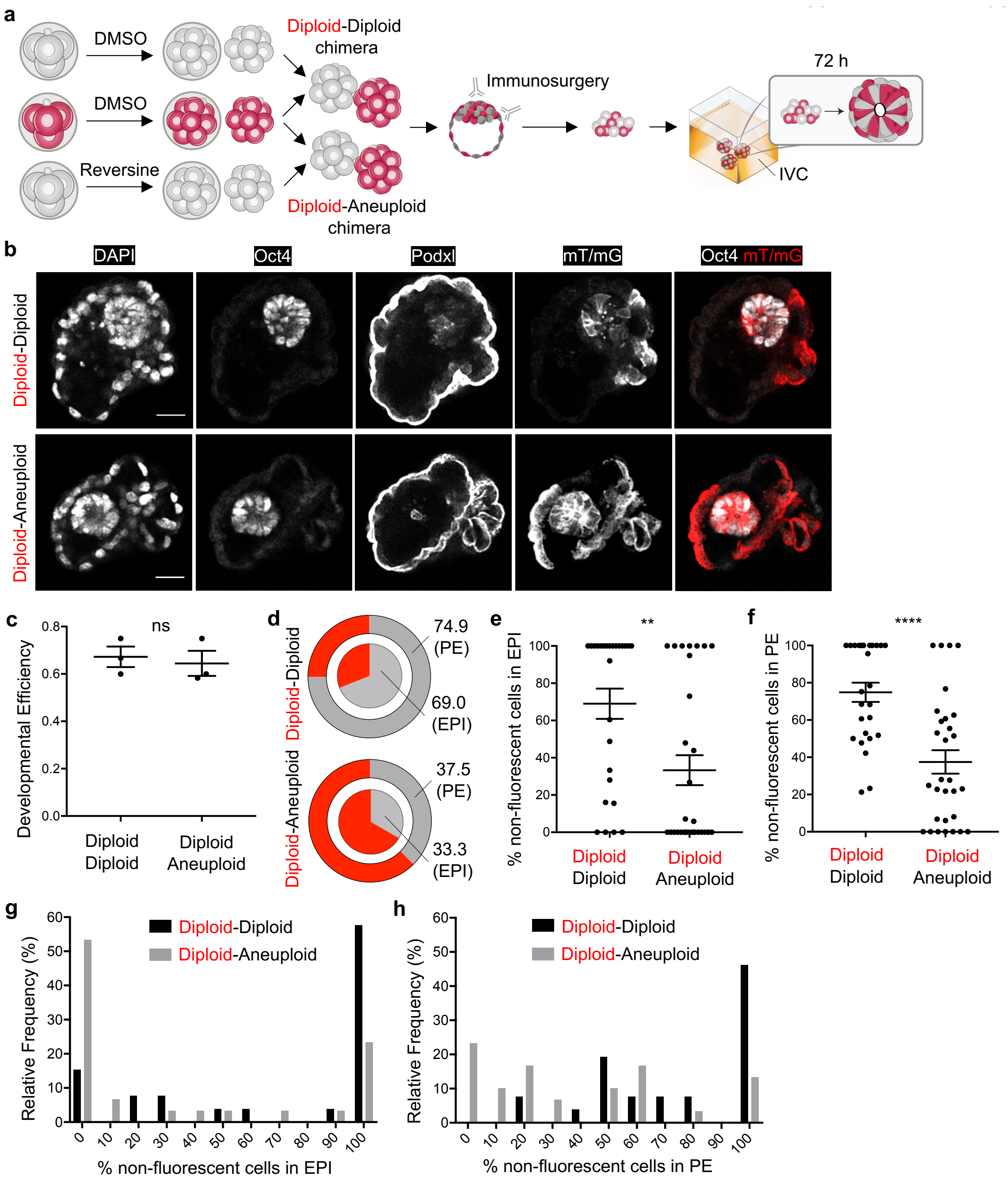

Supplementary Figure 5. Aneuploid cells become eliminated during epiblast remodelling at peri-implantation development of double size mosaic embryos *in vitro*. (**a**) Embryos were treated with reversine (or DMSO) at the 4-8 cell stage transition and 16-cell chimeras containing a 1:1 ratio of control (diploid) and reversine-treated (aneuploid) cells were constructed from mT/mG (red) diploid embryos and non-fluorescent aneuploid embryos at the 8-cell stage. At the late blastocyst stage, immunosurgery was performed to isolate the ICM from the TE. The chimeras were cultured in IVC medium for 72 h to allow their development into an epithelised EPI surrounded by a PE layer with a central lumen. (**b**) Chimeras were assessed for the contribution of aneuploid cells to the EPI and the PE after 72 h *in vitro* culture. In these examples, the diploid-diploid chimera contains both red fluorescent and non-fluorescent cells. Whereas,

the majority of the diploid-aneuploid chimera originates from the red fluorescent diploid clone. Scale bars, 30 µm. (**c**) The efficiency of chimeras in forming an epithelised EPI surrounded by a PE layer with a central lumen after 72 h *in vitro* culture is equivalent for diploid-diploid controls and diploid-aneuploid chimeras. n = 3 independent experimental groups. Mann-Whitney test and ns = not significantly different. (**d**) After culture, average distribution of red fluorescent and non-fluorescent cells was assessed for both types of chimeras in the EPI and PE. Each chimera was assessed for the percentage of non-fluorescent cells to total number of cells in the EPI (**e**) and PE (**f**). Frequency distribution of non-fluorescent cells in the EPI (**g**) and PE (**h**) of control diploid (red)-diploid (non-fluorescent) chimeras and of diploid (red)-aneuploid (non-fluorescent) chimeras. For graph (**e**), Mann-Whitney test and $^{**}p = 0.0018$. For graph (**f**), Student's t-test and $^{****}p < 0.0001$. For graphs (**c**), (**e**) and (**f**), all data are mean ± s.e.m. For all the graphs and (**b**), diploid-diploid n = 26 chimeras; n = 1772 EPI cells; n = 2540 PE cells and diploid-aneuploid n = 30 chimeras; n = 1961 EPI cells; n = 3022 PE cells. Source data are provided as a Source Data file.

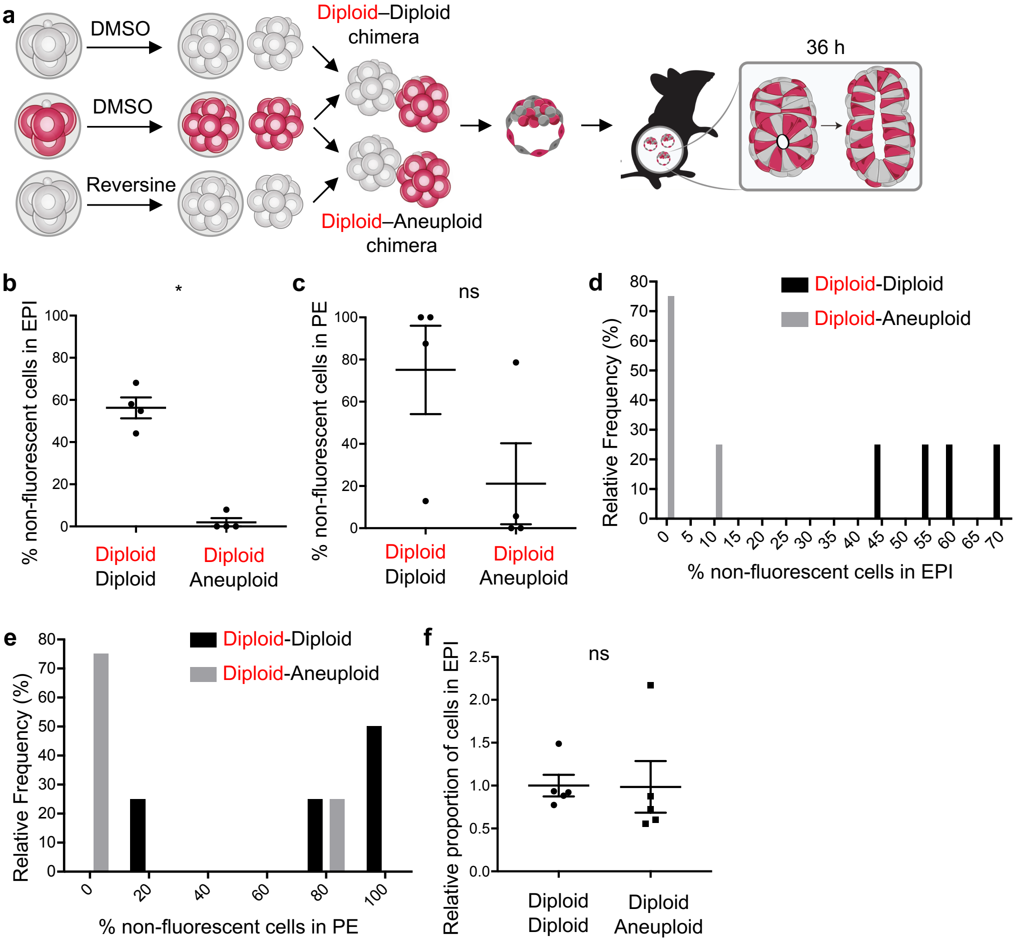

Supplementary Figure 6. Aneuploid cells become eliminated during epiblast remodelling at peri-implantation development of double size mosaic embryos *in vivo*. (**a**) Embryos were treated with reversine (or DMSO) at the 4-8 cell stage transition and 16-cell chimeras containing a 1:1 ratio of control (diploid) and reversine-treated (aneuploid) cells were constructed from mT/mG (red) diploid embryos and non-fluorescent aneuploid embryos at the 8-cell stage. At the early blastocyst stage, chimeras were transferred to pseudo-pregnant mothers and recovered 12 h after implantation and cultured in IVC medium for 36 h. After culture, each chimera was assessed for the percentage of non-fluorescent cells to total number of cells in the EPI (**b**) and PE (**c**). Frequency distribution of non-fluorescent cells in the EPI (**d**) and PE (**e**) of diploid (red)-diploid (non-fluorescent) chimeras and of diploid (red)-aneuploid (non-fluorescent) chimeras. For graphs (**b**), (**c**), (**d**) and (**e**), diploid-diploid n = 4 chimeras; n = 719 EPI cells; n = 938 PE cells and diploid-aneuploid n = 4 chimeras; n = 734 EPI cells; n = 956 PE cells. (**f**) Relative number of cells in the EPI was analysed for both types of chimeras (relative to the average of diploid-diploid chimeras) at the end of the peri-implantation culture. Diploid-diploid n = 5 chimeras; n = 873 EPI cells and diploid-aneuploid n = 5 chimeras; n = 860 EPI cells. For graphs (**b**), (**c**) and (**f**), Mann-Whitney test, ns = not significantly different, *p = 0.0286 and all data are mean ± s.e.m. Source data are provided as a Source Data file.

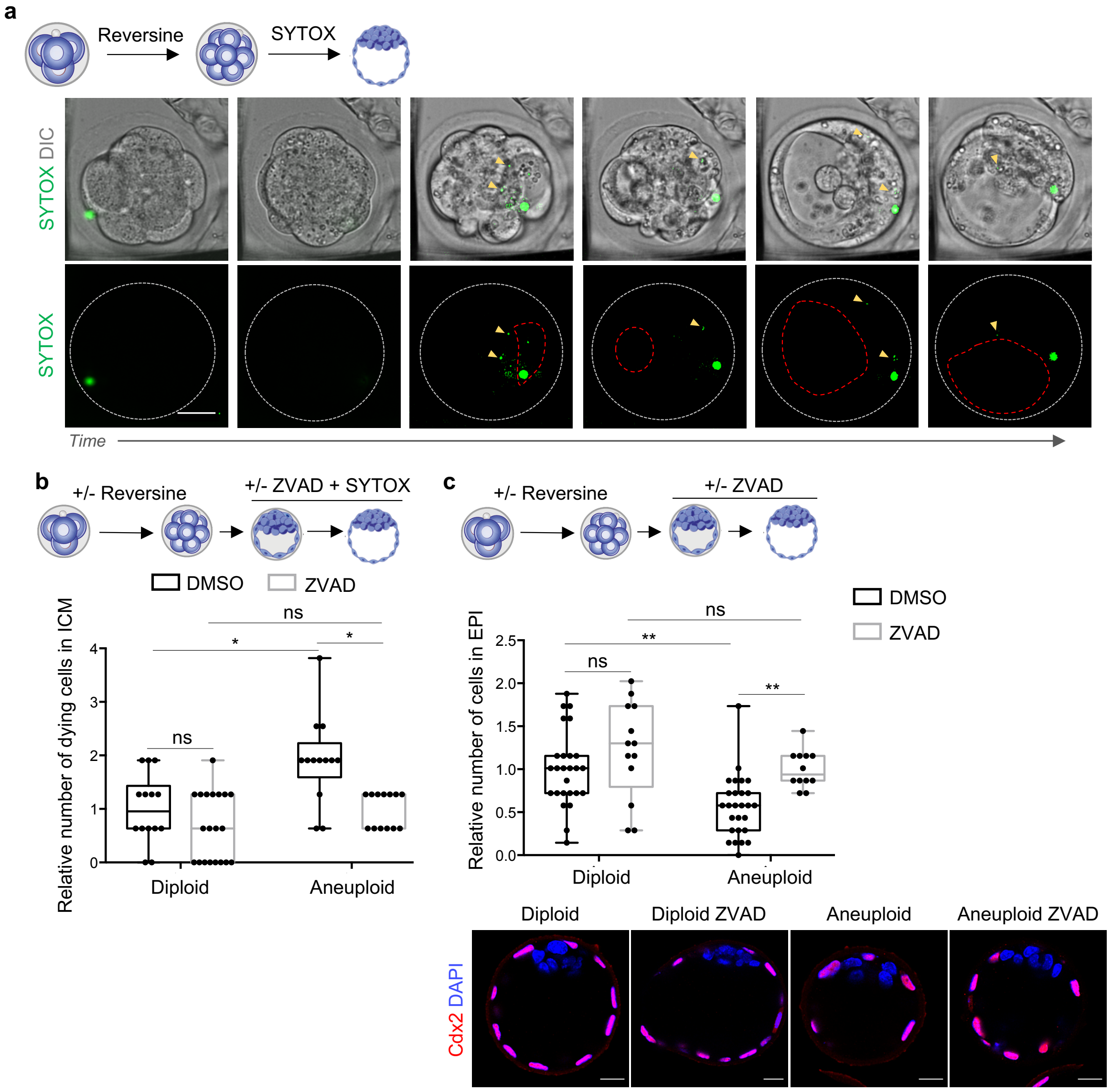

Supplementary Figure 7. Elimination of aneuploid cells from the ICM of aneuploid embryos during blastocyst maturation by apoptosis. (**a**) Embryos were treated with reversine at the 4-8 cell stage transition. Aneuploid (reversine-treated) embryos were imaged in the presence of SYTOX to label dying cells until the late blastocyst stage. Sequential images from a time-lapse recording show dying aneuploid cells (arrows) from the early blastocyst stage onwards. Red line marks the site of the cavity. Scale bar, 30 µm, n = 70 embryos. (**b**) Embryos were treated with reversine (or DMSO) at the 4-8 cell stage transition. Diploid and aneuploid embryos were imaged in the presence of ZVAD or DMSO and SYTOX from the early blastocyst to the late blastocyst stage (24 h). The number of dying cells in the ICM was assessed relative to the average number of dying cells in the ICM in DMSO-treated diploid embryos. Diploid n = 14 embryos, aneuploid n = 13 embryos, diploid ZVAD n = 20 embryos, aneuploid ZVAD n = 13 embryos. (**c**) Embryos were treated with reversine (or DMSO) at the 4-8 cell stage transition. Diploid and aneuploid embryos were cultured in the presence of ZVAD or DMSO during

blastocyst maturation. Scale bars, 20 µm. The EPI cell number was assessed relative to the average EPI cell number in DMSO-treated diploid embryos. Diploid n = 26 embryos; n = 153 EPI cells, aneuploid n = 27 embryos; n = 91 EPI cells, diploid ZVAD n = 13 embryos; n = 95 EPI cells, aneuploid ZVAD n = 12 embryos; n = 71 EPI cells. For graphs (**b**) and (**c**), all data are mean ± s.e.m., Kruskal-Wallis test, ns = not significantly different, *p = 0.0323 (diploid versus aneuploid) and 0.0286 (aneuploid versus aneuploid ZVAD), **p = 0.0017 (diploid versus aneuploid) and 0.0065 (aneuploid versus aneuploid ZVAD). Source data are provided as a Source Data file.

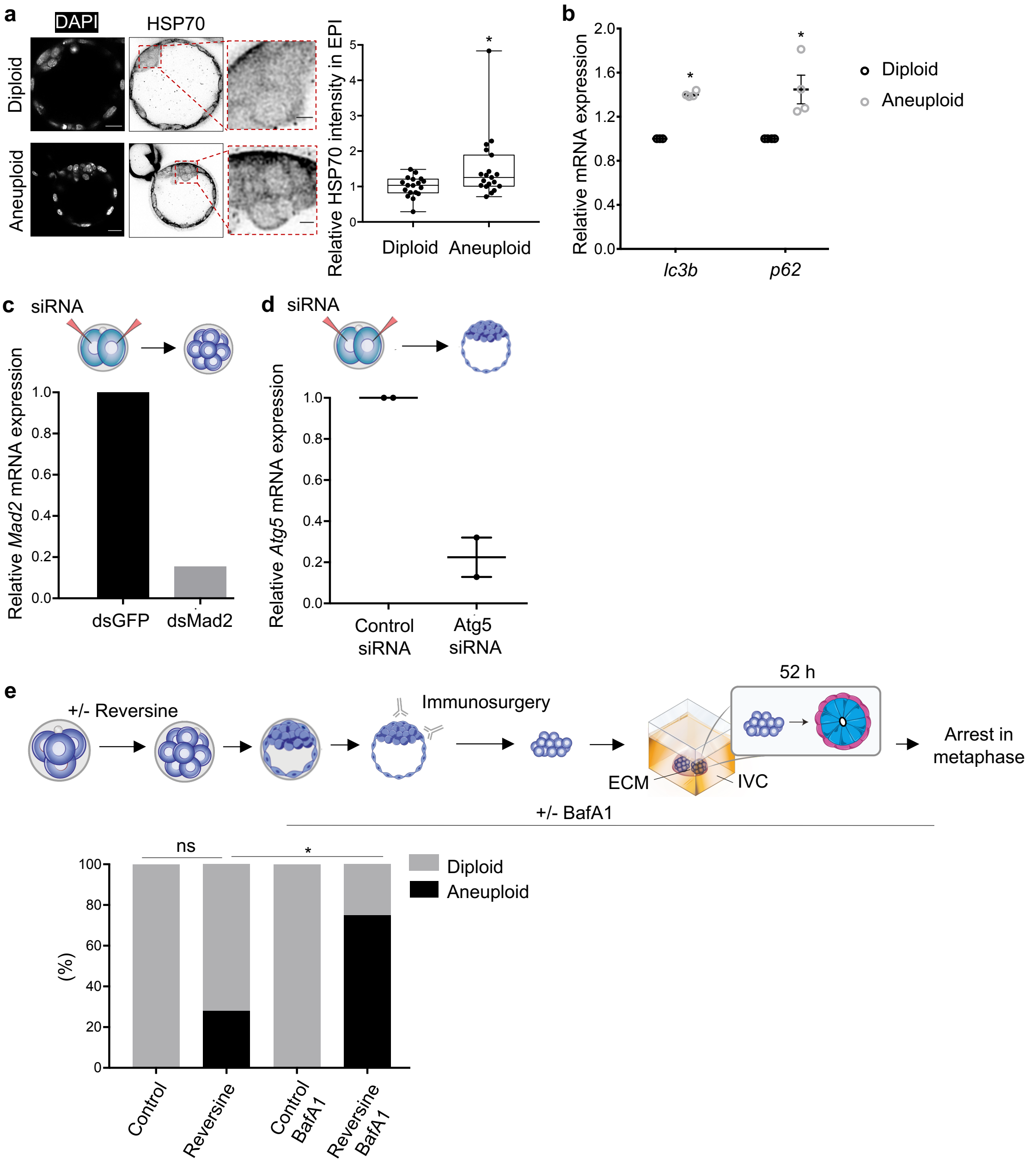

Supplementary Figure 8. The upregulation of autophagy mediates the elimination of aneuploid cells from the ICM. (**a**) The ratio of cystolic HSP70 to DAPI fluorescence intensity was assessed (relative to the average of diploids) in the late blastocyst EPI for diploid (DMSO-treated) and aneuploid (reversine-treated) embryos. Each dot represents relative HSP70 levels in an embryo. Mann-Whitney test, *p = 0.0310. Data are shown as individual data points in a Box and Whiskers graph (bottom: 25%; top: 75%; line: median; whiskers: min to max). Diploid n = 18 embryos and aneuploid n = 19 embryos. Scale bars, 20µm. Squares indicate magnified regions. Scale bars, 5µm. (**b**) mRNA expression for genes involved in autophagy were compared between late blastocyst stage diploid and aneuploid embryos using qRT-PCR. Diploid n = 69 embryos, aneuploid n = 67 embryos. Mann-Whitney test, *p = 0.0286. (**c**) Both blastomeres at the 2-cell stage were injected with dsRNA targeting GFP (dsGFP), as control or Mad2 (dsMad2).

Mad2 mRNA expression was assessed by qRT-PCR at the 8-cell stage (relative to dsGFP-injected embryos). dsGFP n = 15, dsMad2 n = 15 embryos. (**d**) Both blastomeres at the 2-cell stage were injected with siRNA. *Atg5* mRNA expression was assessed by qRT-PCR at the late blastocyst stage (relative to control siRNA-injected embryos). Control n = 30, atg5 siRNA n = 21 embryos. For graphs (**b**), (**c**) and (**d**), all data are mean ± s.e.m. (**e**) Control and reversine-treated embryos were cultured in DMSO or BafA1 from the early to late blastocyst stage. After immunosurgery at the late blastocyst stage, ICMs were embedded in Matrigel and cultured in IVC medium with DMSO or BafA1. Number of chromosomes per cell were assessed by chromosome spread after 52 h of IVC culture. If the number of chromosomes = 40, the cell was categorised diploid and if the number of chromosomes ≠ 40, as aneuploid. Diploid n = 10 cells, aneuploid n = 18 cells, diploid BafA1 n = 10 cells, aneuploid BafA1 n = 8 cells. Fisher's exact test, ns = not significantly different, *p = 0.0384. Source data are provided as a Source Data file.

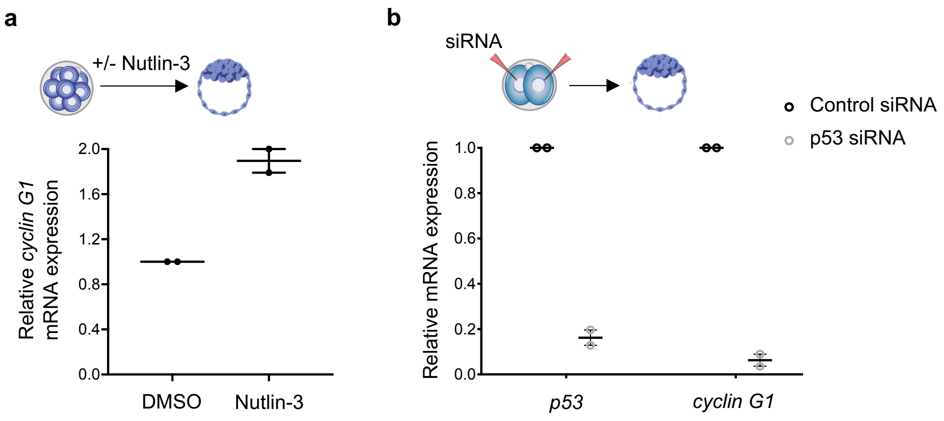

Supplementary Figure 9. Nutlin-3 and p53 siRNA efficiency in pre-implantation embryos. (**a**) Embryos were treated with Nutlin-3 (or DMSO) from the late 8-cell to the late blastocyst stage. mRNA expression for the gene *cyclin G1* was assessed by qRT-PCR at the late blastocyst stage (relative to control embryos). All data are mean ± s.e.m. Control n = 29, Nutlin-3-treated n = 30 embryos. (**b**) Both blastomeres at the 2-cell stage were injected with siRNA. mRNA expression for the genes *p53* and *cyclin G1* was assessed by qRT-PCR at the late blastocyst stage (relative to control siRNA-injected embryos). All data are mean ± s.e.m. Control n = 20, p53 siRNA n = 23 embryos. Source data are provided as a Source Data file.

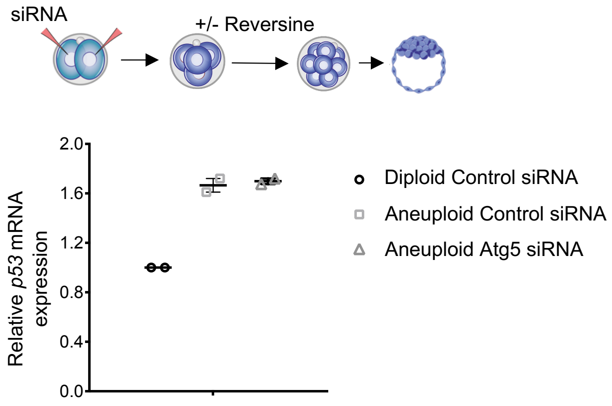

Supplementary Figure 10. Atg5 siRNA injection in aneuploid pre-implantation embryos has no effect on p53 expression. Both blastomeres at the 2-cell stage were injected with siRNA and the embryos were treated at the 4-8 cell stage with reversine or DMSO. *p53* mRNA expression was assessed by qRT-PCR at the late blastocyst stage (relative to control siRNA-injected diploids). Diploid n = 20 embryos, aneuploid n = 21 embryos, aneuploid Atg5 siRNA n = 28 embryos. All data are mean ± s.e.m. Source data are provided as a Source Data file.

Supplementary Table 1. Primer sequences for qRT-PCR

Gene	Forward Sequence – (5' to 3')	Reverse Sequence – (5' to 3')
gapdh	AGAGACGGCCGCATCTTC	CCAATACGGCCAAATCCGT
atg5	GGATGGGACTGCAGAATGACAG	AGCTTCTGGATGAAAGGCCG
lc3b	TTATAGAGCGATACAAGGGGGAG	CGCCGTCTGATTATCTTGATGAG
cyclin G1	TTTTATTTGGCTGTGAAAGCGAC	AGGTCTGAAACCGTGAACCTAT
p53	GTCACAGCACATGACGGAGG	TCTTCCAGATGCTCGGGATAC
p21	CCTGGTGATGTCCGACCTG	CCATGAGCGCATCGCAATC
p62	AGGATGGGGACTTGGTTGC	TCACAGATCACATTGGGGTGC
bcl2	GCTACCGTCGTGACTTCGC	CCCCACCGAACTCAAAGAAGG
mad2	CTGACCCCGAGCTCATAAAGT	ACTGAGCACTTGTACAGCCA
