# Supplementary material for: Autophagy-mediated apoptosis eliminates aneuploid cells in a mouse model of chromosome mosaicism
Source: Nat Commun. 2020 Jun 11;11:2958. doi: 10.1038/s41467-020-16796-3 (PMC7290028; doi:10.1038/s41467-020-16796-3)
Supplement: Supplementary file 3 — Reporting Summary [file 41467_2020_16796_MOESM3_ESM.pdf]

nature research

Corresponding author(s): Magdalena-Zernicka-Goetz

Last updated by author(s): Feb 27, 2020

Reporting Summary

Nature Research wishes to improve the reproducibility of the work that we publish. This form provides structure for consistency and transparency in reporting. For further information on Nature Research policies, see Authors & Referees and the Editorial Policy Checklist .

Statistics

For all statistical analyses, confirm that the following items are present in the figure legend, table legend, main text, or Methods section.

n/a	Confirmed	
☐	☒	The exact sample size (*n*) for each experimental group/condition, given as a discrete number and unit of measurement
☐	☒	A statement on whether measurements were taken from distinct samples or whether the same sample was measured repeatedly
☐	☒	The statistical test(s) used AND whether they are one- or two-sided<br>*Only common tests should be described solely by name; describe more complex techniques in the Methods section.*
☒	☐	A description of all covariates tested
☐	☒	A description of any assumptions or corrections, such as tests of normality and adjustment for multiple comparisons
☐	☒	A full description of the statistical parameters including central tendency (e.g. means) or other basic estimates (e.g. regression coefficient) AND variation (e.g. standard deviation) or associated estimates of uncertainty (e.g. confidence intervals)
☐	☒	For null hypothesis testing, the test statistic (e.g. *F*, *t*, *r*) with confidence intervals, effect sizes, degrees of freedom and *P* value noted<br>*Give P values as exact values whenever suitable.*
☒	☐	For Bayesian analysis, information on the choice of priors and Markov chain Monte Carlo settings
☒	☐	For hierarchical and complex designs, identification of the appropriate level for tests and full reporting of outcomes
☒	☐	Estimates of effect sizes (e.g. Cohen's *d*, Pearson's *r*), indicating how they were calculated

Our web collection on statistics for biologists contains articles on many of the points above.

Software and code

Policy information about availability of computer code

Data collection	n/a
Data analysis	For graphical statistics and statistical tests , GraphPad Prism 7.0a was used. ImageJ software (version: 2.0.0-rc-69/1.52p) was used for image processing and analysis. SlideBook Software 6.0.4 (24366) Intelligent Imaging Innovations was used for video processing.

For manuscripts utilizing custom algorithms or software that are central to the research but not yet described in published literature, software must be made available to editors/reviewers. We strongly encourage code deposition in a community repository (e.g. GitHub). See the Nature Research guidelines for submitting code & software for further information.

Data

Policy information about availability of data

All manuscripts must include a data availability statement. This statement should provide the following information, where applicable:

- Accession codes, unique identifiers, or web links for publicly available datasets
- A list of figures that have associated raw data
- A description of any restrictions on data availability

The authors claim that all relevant data of the findings in this work are provided within the paper and Supplementary Information files. Raw data are provided in the Source Data File.

Field-specific reporting

Please select the one below that is the best fit for your research. If you are not sure, read the appropriate sections before making your selection.

Life sciences study design

All studies must disclose on these points even when the disclosure is negative.

Sample size	Sample size was determined based on our previous experience and the work of other groups using mouse embryos as experimental model systems. No statistical method was used to predetermine sample size.<br>Nature Communications 7, 11165 (2016)<br>Nature Communications 4, 2251 (2013).
Data exclusions	Embryos that showed apparent developmental delay or cell death were discarded. This was done based on our previous experience: Nature Communications 7, 11165 (2016) and Nature Communications 8, 921 (2017).
Replication	In the study, all attempts at replication were successful.<br>Each result described in the paper is based on at least two independent biological replicates (except for the experiment in Suppl. Fig. 8c) but very often an experiment is based on more than two experiments. However, the experiment in Suppl. Fig. 8c has three technical replicates.
Randomization	Samples (mouse embryos) were allocated randomly into experimental groups
Blinding	The investigators were not blinded to group allocation during experiments and outcome assessment due to the experimental design in which embryos had to undergo specific drug treatments. Therefore blinding was not possible.

Reporting for specific materials, systems and methods

We require information from authors about some types of materials, experimental systems and methods used in many studies. Here, indicate whether each material, system or method listed is relevant to your study. If you are not sure if a list item applies to your research, read the appropriate section before selecting a response.

Materials & experimental systems

n/a	Involved in the study	
☐	☒	Antibodies
☒	☐	Eukaryotic cell lines
☒	☐	Palaeontology
☐	☒	Animals and other organisms
☒	☐	Human research participants
☒	☐	Clinical data

Methods

n/a	Involved in the study	
☒	☐	ChIP-seq
☒	☐	Flow cytometry
☒	☐	MRI-based neuroimaging

Antibodies

Antibodies used	Primary antibodies used: mouse anti-Cdx2 (Biogenex, 1:200), mouse anti-Oct3/4 (Santa Cruz Biotechnology, 5279, 1:200), goat Gata4 (Santa Cruz Biotechnology, 1237, 1:200), rat anti-Podocalyxin (R&D Systems, MAB1556, 1:500), rabbit anti-RFP (Rockland, 600-401-379, 1:500), mouse anti-HSP70 (Proteintech, 66183, 1:50), rabbit anti-p62 (Proteintech, 55274, 1:100), rabbit anti-phospho-Histone H3 (Millipore, 06-570, 1:200), and rabbit anti-LC3B (Cell Signalling, 2775, 1:200). Also, Alexa Fluor secondary antibodies (Thermo Fisher Scientific, 1:400) used.
Validation	Primary Antibodies used in whole mount immunofluorescence:<br>Oct3/4, RFP and Podocalyxin: correct localisation after staining as shown in Figure 1b<br>Cdx2: correct localisation after staining as shown in reference 14<br>Gata4: correct localisation after staining as shown in Supplementary Figure 2c<br>phospho-Histone H3: correct localisation after staining as shown in Figure 3d<br>HSP70: validation on manufacturer's website after staining of HEK-293 cells, as reported elsewhere (BMC Cancer 14, 639 (2014))<br>p62: validation on manufacturer's website where HepG2 cells showed more p62 puncta when starved<br>LC3B: validation on manufacturer's website where HCT-116 cells showed more LC3B puncta after choroquine treatment

Animals and other organisms

Policy information about studies involving animals; ARRIVE guidelines recommended for reporting animal research

Laboratory animals	Mice (Mus musculus) were used to obtain mouse embryos for this study. 4- to 6-week-old F1 (C57Bl/6 x CBA) females with F1 (C57Bl/6 x CBA) males or with Histone H2B-GFP or mT/mG males. Animals were maintained in the Animal Facility at 12:12 light cycle, 21-23 degrees, 55% +/- 10% humidity and provided with food and water ad libitum.
Wild animals	The study did not involve wild animals.

Field-collected samples	The study did not involve samples collected from the field.
Ethics oversight	All experiments involving mice have been regulated by the Animals (Scientific Procedures) Act 1986 Amendment Regulations 2012 and additional ethical review by the University of Cambridge Animal Welfare and Ethical Review Body (AWERB). Experiments were authorised by the Home Office (Licence number: 70/8864).

Note that full information on the approval of the study protocol must also be provided in the manuscript.
