# Supplementary material for: Autophagy-mediated apoptosis eliminates aneuploid cells in a mouse model of chromosome mosaicism
Source: Nat Commun. 2020 Jun 11;11:2958. doi: 10.1038/s41467-020-16796-3 (PMC7290028; doi:10.1038/s41467-020-16796-3)
Supplement: Supplementary file 8 — Description of Additional Supplementary Files [file 41467_2020_16796_MOESM8_ESM.pdf]

Title: Supplementary Movie 1:
Description: Diploid-aneuploid mosaic chimera (8-cell chimera generated at the 8-cell stage, diploid cells are labelled with red membrane mT/mG and aneuploid cells with H2B-GFP), cultured in vitro showing apoptosis of an aneuploid cell during pre- to post-implantation development, Related to Figure 2a.

Title: Supplementary Movie 2:
Description: Diploid-aneuploid mosaic chimera (16-cell chimera generated at the 8-cell stage, diploid cells are labelled with red membrane mT/mG and aneuploid cells with H2B-GFP), cultured in vitro showing apoptosis of an aneuploid cell during pre- to post-implantation development, Related to Figure 2b.

Title: Supplementary Movie 3:
Description: Diploid-aneuploid mosaic chimera (8-cell chimera generated at the 8-cell stage, diploid cells are labelled with red membrane mT/mG and aneuploid cells with H2B-GFP), which underwent implantation in vivo showing apoptosis of an aneuploid cell in the epiblast during early post-implantation development, Related to Figure 2c.

Title: Supplementary Movie 4:
Description: Pre-implantation aneuploid embryo development from the late 8-cell stage to the late blastocyst stage in vitro in the presence of SYTOX Orange Dead Cell Stain, Related to Supplementary Figure 7a.
